# Feasibility of preoperative ^125^I seed-guided tumoural tracer injection using freehand SPECT for sentinel lymph node mapping in non-palpable breast cancer

**DOI:** 10.1186/s13550-014-0019-5

**Published:** 2014-05-03

**Authors:** Bas Pouw, Linda J de Wit-van der Veen, Daan Hellingman, Oscar R Brouwer, Marie-Jeanne TFD Vrancken Peeters, Marcel PM Stokkel, Renato A Valdés Olmos

**Affiliations:** 1 MIRA Institute for Biomedical Technology and Technical Medicine, University of Twente, Enschede 7500 AE, The Netherlands; 2 Department of Nuclear Medicine, The Netherlands Cancer Institute, Antoni van Leeuwenhoek Hospital, Amsterdam 1066 CX, The Netherlands; 3 Department of Surgical Oncology, The Netherlands Cancer Institute, Antoni van Leeuwenhoek Hospital, Amsterdam 1066 CX, The Netherlands

**Keywords:** Freehand SPECT, Declipse®SPECT, 125Iodine seed, Sentinel lymph node biopsy, Non-palpable breast cancer

## Abstract

**Background:**

This study was designed to explore the feasibility of replacing the conventional peri-/intratumoural ultrasound (US)-guided technetium-99m albumin nanocolloid (^99m^Tc-nanocolloid) administration by an injection of the same tracer guided by a freehand single-photon emission computed tomography (SPECT) device in patients with non-palpable breast cancer with an iodine-125 (^125^I) seed as tumour marker, who are scheduled for a sentinel lymph node biopsy (SLNB). This approach aimed to decrease the workload of the radiology department, avoiding a second US-guided procedure.

**Methods:**

In ten patients, the implanted ^125^I seed was primarily localised using freehand SPECT and subsequently verified by conventional US in order to inject the ^99m^Tc-nanocolloid. The following 34 patients were injected using only freehand SPECT localisation. In these patients, additional SPECT/CT was acquired to measure the distance between the ^99m^Tc-nanocolloid injection depot and the ^125^I seed. In retrospect, a group of 21 patients with US-guided ^99m^Tc-nanocolloid administrations was included as a control group.

**Results:**

The depth difference measured by US and freehand SPECT in ten patients was 1.6 ± 1.6 mm. In the following 36 ^125^I seeds (34 patients), the average difference between the ^125^I seed and the centre of the ^99m^Tc-nanocolloid injection depot was 10.9 ± 6.8 mm. In the retrospective study, the average distance between the ^125^I seed and the centre of the ^99m^Tc-nanocolloid injection depot as measured in SPECT/CT was 9.7 ± 6.5 mm and was not significantly different compared to the freehand SPECT-guided group (two-sample Student's *t* test, *p* = 0.52).

**Conclusion:**

We conclude that using freehand SPECT for ^99m^Tc-nanocolloid administration in patients with non-palpable breast cancer with previously implanted ^125^I seed is feasible. This technique may improve daily clinical logistics, reducing the workload of the radiology department.

## Background

The use of mammographic screening in nationwide programs within western countries has led to an increase in the number of women with non-palpable breast cancer lesions [1,2]. Currently, more than 25% of the radiological suspicious breast lesions are considered clinically non-palpable [3]. Accordingly, in many patients, accurate pre- and intraoperative localisation of these non-palpable lesions is important for adequate breast-conserving surgery. At present, four different techniques are used to localise the tumour prior to excision: wire-, ultrasound (US)-, carbon- and radio-guided (i.e. guided by a radionuclide) localisation [3-5]. When lymph node involvement is undetermined, these approaches are combined with a sentinel lymph node (SLN) procedure [6,7].

At The Netherlands Cancer Institute, both radio-guided occult lesion localisation (ROLL) with radioactive technetium-99m albumin nanocolloid (^99m^Tc-nanocolloid) and radioactive seed localisation (RSL) are used for non-palpable breast tumour localisation during surgery [8]. In the case of RSL, a 3.7 to 10.7 MBq iodine-125 seed (^125^I) with a half-life time of 60 days is preoperatively implanted into the malignancy using US guidance in most cases. When the tumour was only visible on mammography, placement of the ^125^I seed was performed under stereotactic guidance. In our institute, the location of the ^125^I seed is always confirmed by an additional mammogram at the day of implantation. Recent studies show advantages when looking at resection margins, duration of localisation and surgical excision time for RSL or ROLL over wire-based localisation [3],[9-11]. At The Netherlands Cancer Institute, RSL is a standard procedure, and over 1,000 ^125^I seeds have been implanted since 2008.

In all patients scheduled for tumour excision, the procedure is combined with sentinel lymph node biopsy (SLNB) for regional staging of the disease. This staging is of great significance for patient outcome, being a predictor of presence for further metastasis in the axillary basin [12]. Clinical protocols for this procedure may vary between institutes because the radiocolloid injection site for SLNB is still a matter of controversy [13-17]. At The Netherlands Cancer Institute, the ^99m^Tc-nanocolloid for SLNB in non-palpable breast cancer is preferably administered intratumourally by US guidance, although, in clinical practice, it turns out to be either peri- or intratumoural. Peritumoural is defined as the area at least within a radius of 10 mm to the tumour border. Intratumoural injections can sometimes result in resistance of the tumour tissue while administering the tracer; this is solved by small injection volumes (<0.2 ml) and to slowly pull a little bit back while administering the tracer. This peri- or intratumoural radiopharmacologic administration will result in extra-axillary SLNs, which, in our institute, are included for diagnosis [18]. Prior to surgery, the radiologist localises the ^125^I seed by ultrasonic reflection of the titanium capsule in order to place a needle into the tumour. Subsequently, a nuclear physician injects the ^99m^Tc-nanocolloid [19]. This can be a challenging intervention due to difficulties in localising the ^125^I seed in pathological and irregular breast tissue. Furthermore, the procedure requires two medical specialists (e.g. a radiologist and a nuclear physician) and a technologist. By avoiding this additional US procedure, the workload of the radiology department will be decreased.

Recently, a novel freehand single-photon emission computed tomography (SPECT) system (declipse**®**SPECT, SurgicEye GmbH, Munich, Germany) for three-dimensional (3D) radio-guided imaging and navigation has been introduced. This device combines a conventional gamma probe with an optical tracking system. An algorithm links the measured counts from the location of the gamma probe in space and, accordingly, reconstructs a 3D visualisation [20,21]. The purpose of this study is to validate ^125^I seed localisation guided by freehand SPECT in patients with non-palpable breast cancer in order to facilitate ^99m^Tc-nanocolloid injections. The results of this study could also serve as a proof of concept for use of this specific radio-guided navigation technique in other malignancies.

## Methods

### Patient population

Forty-four patients with a peri-/intratumoural ^125^I seed (STM1251, Bard Brachytherapy, Inc., Carol Stream, IL, USA) and scheduled for an SLN procedure were included. The patients were included in consecutive order, and inclusion was based on availability of researchers, SPECT/CT scanner and the freehand SPECT device. The study protocol included a group of patients scheduled for both US and freehand SPECT (group 1, *n* = 10) followed by a second group of patients investigated with only the freehand SPECT probe (group 2, *n* = 34). The results of the second group were compared with a control group of patients who had received injections guided by US (group 3, *n* = 21). Experienced radiologists measured the tumour size by means of MRI or mammography. The characteristics of the groups are as follow:

 Group 1: In ten patients in the period from October 2012 to December 2012, the location and depth of the ^125^I seed were measured using both freehand SPECT and US. The ^99m^Tc-nanocolloid was injected exactly according to the standard protocol based on US-guided injections, which is the technique of choice at the Netherlands Cancer Institute. The standard protocol means no additional SPECT/CT scan to limit additional radiation exposure for patients.

 Group 2: In the period from December 2012 to April 2013, 34 patients were included. The location and depth of the ^125^I seed were measured using freehand SPECT followed by a freehand SPECT-guided injection with ^99m^Tc-nanocolloid. These 34 patients received a SPECT/CT scan to measure the accuracy of the ^99m^Tc-nanocolloid injection location in relation to the ^125^I seed.

 Group 3: The control group was constituted retrospectively by 21 consecutive patients who underwent US-guided ^99m^Tc-nanocolloid injection near the ^125^I seed and a SPECT/CT in the period from April 2012 to March 2013. This means that only those patients who had received a SPECT/CT in the context of a standard SLN procedure (i.e. non-visualisation of lymphatic drainage or aberrant lymphatic drainage on the planar imaging) were included. This group is selected to study the US-guided injection depots of ^99m^Tc-nanocolloid by means of SPECT/CT without making additional SPECT/CT scans.

### The standard clinical SLN protocol

All patients who are to undergo a SLNB get a ^99m^Tc-nanocolloid (GE Healthcare, Eindhoven, The Netherlands) injection of 140 MBq in 0.2 ml 1 day prior to surgery. Five-minute static scintigraphic images are acquired from the anterior and lateral sides 5 to 30 min and 2 to 3 h post-injection, respectively. In case of non-visualisation or aberrant lymphatic drainage, an additional SPECT/CT scan is obtained. All SPECT/CT data are acquired using a hybrid camera (Symbia T, Siemens, Erlangen, Germany). The dual-head SPECT (128 × 128 matrix, 40 frames, 30 s/frame) is performed using 9° angular steps in a 30-s time frame. For CT (130 kV, 40 mA, B30s kernel), 5-mm slices are obtained. Both attenuation and scatter correction are applied.

### Freehand SPECT acquisition and reconstruction

**Figure 1:**
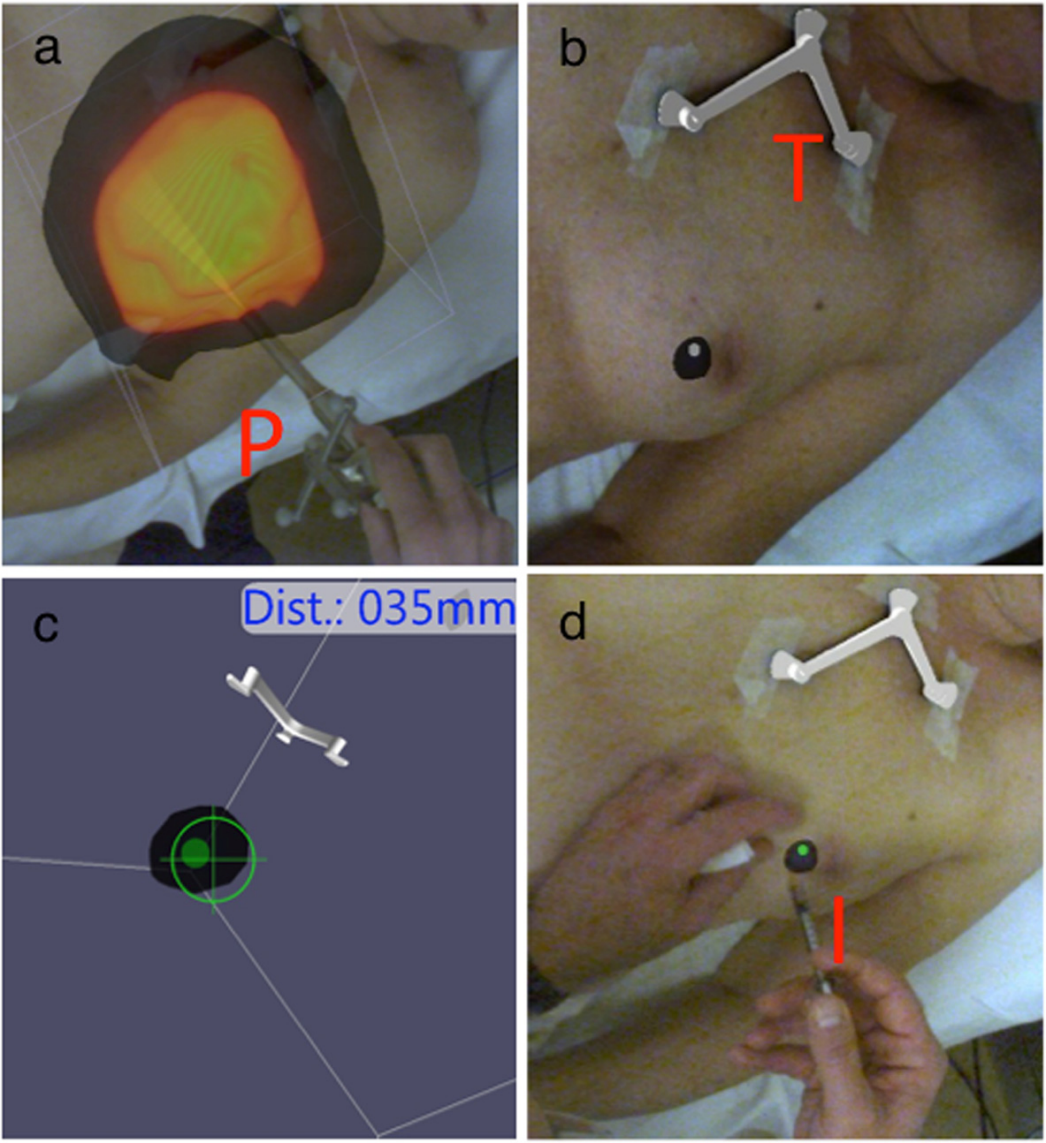
**Freehand SPECT method. (a)** Data acquisition using the freehand SPECT device; radioactivity is measured with the probe from multiple directions. *P* is the probe, and in yellow, the detection beam of the probe. The orange cloud is an accumulation of the area where it is measured. **(b)** The localisation of the ^125^I seed after reconstruction. The ^125^I seed reconstruction is projected over the optical image in purple. *T* is the patient tracker. **(c)** 3D visualisation of the distance and direction of the probe tip to the ^125^I seed. **(d)** Injection of ^99m^Tc-nanocolloid guided by freehand SPECT. *I* is the tracer injection localisation.

This method was based on combining counts measured with a conventional gamma probe with data of the location and orientation of the gamma probe using a reflective reference target attached to a specific site on the probe. Through a calibration procedure, the relation between the gamma probe tip and the reference target was determined [22]. To acquire an accurate 3D volume reconstruction from the count data, a surface scan was made by hovering the probe over the area of interest in three different orientations (e.g. *x*, *y* and *z* planes). The system requires at least 1,500 measurements to accurately create a 3D visualisation; in our protocol, we adopted thus a minimum of 2,000 measurements in three or more directions. This planar surface scanning takes about 2 min, and the reconstruction of the volume takes another 20 s. After the reconstruction, the optical camera of the used system was combined with a radioactivity map (Figure 1b). The window level was adjusted by using a touch screen to set a visualisation threshold similar to the ones used in conventional nuclear medicine until the number of hot spots equals the number of ^125^I seeds *in situ*. The 3D window enabled the best navigation to the ^125^I seed (Figure 1c).

For patients of group 1, the perpendicular distance from the skin to the ^125^I seed was determined with the freehand SPECT, and the most intensive focus in the 3D reconstruction was marked on the skin of the patient. Next, the radiologist, who was blinded for the depth information, localised the ^125^I seed with US and measured the perpendicular depth from the marked place on the skin to the ^125^I seed. After which, he injected the ^99m^Tc-nanocolloid close to the ^125^I seed. To avoid breast tissue deformations, it is important that the patient does not change position during the freehand SPECT and US measurements.

For patients of group 2, the perpendicular depth to the ^125^I seed, which was used for US-guided injections as well, was measured per patient, and the optimal injection location was marked on the skin. The needle was injected to the depth indicated by the freehand SPECT (Figure 1c). Three hours after injection of the ^99m^Tc-nanocolloid, a SPECT/CT scan was obtained. Verification of the ^99m^Tc-nanocolloid injection relative to the ^125^I seed was performed by comparing the ^99m^Tc-nanocolloid depot on the SPECT images to the location of the ^125^I seed on the CT scan. The distance from the ^125^I seed to the centre of the activity depot in the axial plane was measured. All distances on SPECT/CT were once more determined by a second independent blinded observer to study the limits of agreement.

### Statistics for data analysis

Continuous variables were represented by mean ± standard deviation (SD). Differences between the measured depths of the ^125^I seed by freehand SPECT and US are evaluated by Bland-Altman graphs. The limits of agreement between the different observers were also evaluated by Bland-Altman graphs. This results in mean difference and 95% confidence interval (95% CI) [23].

## Results

### Group 1: US validation

**Figure 2:**
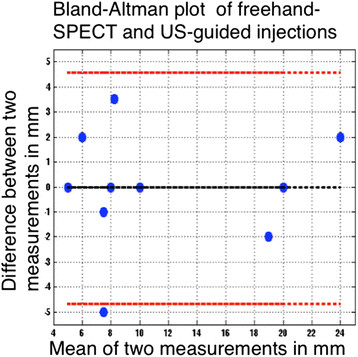
**Bland-Altman analysis for the distances in depth measured with US probe and with the freehand SPECT.** The analysis indicates, with the black broken line, the average of the measurements. The upper and lower red broken lines represent the Bland-Altman limits within the 95% confidence interval.

The characteristics of all ten patients are outlined in Table 1. The lesions were found on various locations in the breast. The ten perpendicular measurements with US and freehand SPECT of the ^125^I seed resulted in absolute variations in the range from 0 to 5 mm. The average difference in depth was 0.05 ± 2.4 mm (range −3.5 to 5 mm), and the absolute average was 1.6 ± 1.6 mm (range 0 to 5 mm). These data are displayed in a Bland-Altman plot, which visualises the mean and the 1.96-time standard deviation ranges [24] (Figure 2).

**Table 1:** Patient information for US validation, patient information for SPECT/CT validation and retrospective US-guided injections

**Parameter**	**Group 1, US validation (*n*** **= 10) (SD, range)**	**Group 2, SPECT validation (*n*** **= 34) (SD, range)**	**Group 3, retrospective US-guided injections (*n*** **= 21) (SD, range)**
Patient age (years)	51 (8.4, 42 to 66)	61.3 (12.1, 26 to 89)	59 (10.6, 42 to 86)
Tumour size (mm)	11.5 (3.1, 9 to 20)	17.1 (13.8, 3 to 60)	18.4 (13.1, 8 to 55)
Tumour type	DCIS (*n* = 3), IDC (*n* = 6), ILC (*n* = 1)	DCIS (*n* = 12), LCIS (*n* = 1), IDC (*n* = 14), ILC (*n* = 4), unknown (*n* = 3)	DCIS (*n* = 14), LCIS (*n* = 4), unknown (*n* = 3)
Number of ^125^I seeds	1	with 1 seed (30), with 2 seeds (4)	1
Location of ^125^I seeds	3 medial, 6 lateral, 1 central	7 medial, 22 lateral, 5 central	5 medial, 13 lateral, 3 central
Days after ^125^I seed implantation	30 (13, 12 to 56)	33.5 (23.3, 10 to 118)	Not measured
^125^I seed depth by US (mm)	11.6 (6.4, 5 to 23)	Not measured	Not measured
^125^I seed depth by freehand SPECT (mm)	11.5 (6.6, 5 to 25)	15.3 (6.7, 8 to 35)	Not measured
Difference in localisation or location (mm)	Mean difference, 0.05 (2.4, −3.5 to 5)		
Absolute mean difference, 1.6 (1.6, 0 to 5)	10.9 (6.8, 0 to 29) (CT compared with SPECT)	9.7 (6.5, 2 to 30) (CT compared with SPECT)	
Irradical procedures	1/10 (focal irradical)	9/34 (focal irradical or irradical)	5/21 (focal irradical or irradical)

DCIS, ductal carcinoma *in situ*; IDC, invasive ductal carcinoma; ILC, invasive lobular carcinoma; LCIS, lobular carcinoma *in situ*.

### Group 2: SPECT/CT validation

The characteristics of all 34 patients are outlined in Table 1. The patients had either one or two ^125^I seeds implanted, and the tumours were located on various locations within the breast. The average distance from the centre of the ^99m^Tc-nanocolloid depot to the ^125^I seed on SPECT/CT was 10.9 ± 6.8 mm (range 0 to 29 mm).

Retrospective analysis of the data showed a possible relation between the number of measurements made by the freehand SPECT and the distance between the ^99m^Tc-nanocolloid injection depot and the ^125^I seed. In ten injections, we noticed that the number of measurements was more than the protocolled 2,000 to 2,500 but was 3,000 to 3,500 measurements at least. We evaluated the differences to study whether a higher number of measurements will result in more accurate injections. The ten injections with more measurements resulted, after measuring the distance between the ^99m^Tc-nanocolloid injection depot and the ^125^I seed, in a mean distance of 10.0 mm instead of 11.2 mm in the other 26 injections.

### Group 3: control group

**Figure 3:**
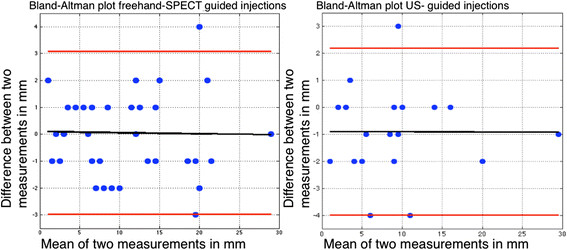
**Bland-Altman analysis for the interobserver agreement between freehand SPECT-guided and US-guided injections.** The analysis indicates, with the black broken line, the average of the measurements. The upper and lower red broken lines represent the Bland-Altman limits within the 95% confidence interval.

**Figure 4:**
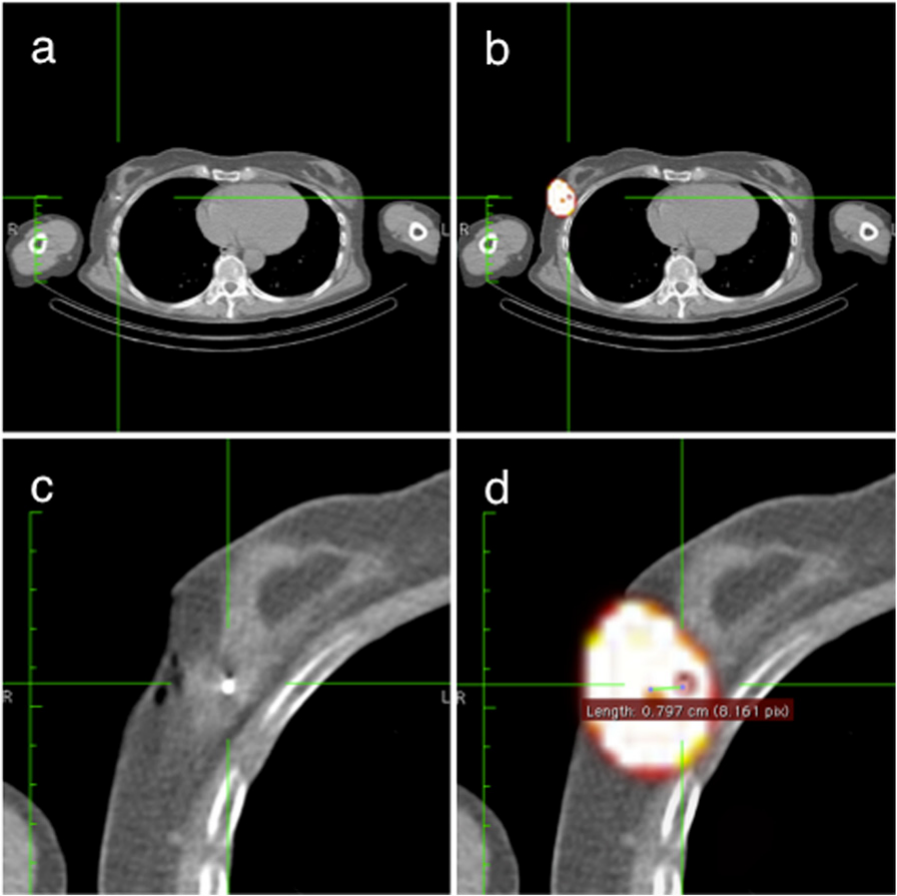
**Axial SPECT/CT images. (a)** The low-dose CT. **(b)** The SPECT and CT images fused. **(c)** Close-up of the low-dose CT. **(d)** The SPECT and CT images fused with the measurement of distance from the centre of activity of the ^99m^Tc-nanocolloid to the ^125^I seed; the measured distance is 8 mm. The green lines indicate the same position in the different images.

In the retrospectively selected US-guided ^99m^Tc-nanocolloid injections (patient group 3), the distance from the depot to the ^125^I seed was 9.7 ± 6.5 mm (range 2 to 30 mm) on SPECT/CT. Comparison of the distance from the depot to the ^125^I seed in the freehand SPECT- (group 2) and US-guided injections (group 3) revealed no significant difference (two-sample Student's *t* test, *p* = 0.52). This means there is no difference in accuracy for US-guided and freehand SPECT-guided injections. The mean difference between the two observations in this setting was 0.5 mm (95% CI 2.9 to −3.5 mm); for freehand SPECT-guided injections, the difference was 0.1 mm (95% CI −3.0 to 3.1 mm), and for the US-guided injections, the difference was 0.9 mm (95% CI −2.2 to 4.0 mm) (Figures 2 and 3). There are images included in this work that illustrate the location of the ^125^I seed and the ^99m^Tc-nanocolloid depot by a fusion of the SPECT signal and the CT scan (Figure 4).

In total, 65 peri-/intratumoural ^99m^Tc-nanocolloid injections were included in this study for analysis. The overall sentinel node (SN) identification rate was 1.2 SNs/patient, and 56/65 had SN visualisation on either lymphoscintigraphy or SPECT/CT. The intraoperative SN identification rate was higher, thanks to prolongation of the time interval (allowing further lymph drainage) and the use of blue dye.

## Discussion

This study demonstrates that peri-/intratumoural ^99m^Tc-nanocolloid injections using a freehand SPECT device are feasible in patients with non-palpable breast cancer marked with a ^125^I seed. The freehand SPECT is able to localise the ^125^I seed and obtains navigation parameters for subsequent SLN procedure-related tracer injection. The manufacturer specified a spatial resolution of 5 mm for the freehand SPECT, suggesting that this device was appropriate for the intervention described in our study [25]. Our results confirmed this by showing a mean difference of 1.6 ± 1.6 mm (range 0 to 5 mm) compared to the conventional US technique. Additionally, it was concluded that the concordance of freehand SPECT-guided administrations compared to US-guided administrations validated by means of SPECT/CT imaging was clinically acceptable for the approach that we pursue. This study was not designed to study a learning curve; we also did not find a learning curve in this limited number of cases. This might be the result of varying observers. However, to use the freehand SPECT device, a training period is required. The results of this study and the benefits of using this technique seem to support the use of freehand SPECT for ^125^I seed-guided radiocolloid injections in patients scheduled for SLNB and thereby enhance the logistics and workload of nuclear medicine and radiology departments.

### Image-guided injections

For SLNB, US-guided injections are commonly used to deliver ^99m^Tc-nanocolloid into or in the vicinity of the tumour [19]. In cases where the ^125^I seed is not identifiable, a stereotaxic procedure is performed. The US-guided injections and the stereotaxic procedures have certain drawbacks; first of all, the planning is more complicated because there are two departments involved, and the time per procedure is variable (15 to 45 min). Furthermore, the localisation of the ^125^I seed may be time consuming and requires a radiologist. The injection using freehand SPECT is straightforward and, as described in the present study, clinically applicable. The procedure can be performed in a nuclear medicine department, which does increase the flexibility in planning. In our experience, the procedure never exceeded 20 min, taking, in average, 10 to 15 min. A second benefit is that this procedure may avoid potential pitfalls in misjudging the identity of the ^125^I seed and thereby an incorrect injection location of ^99m^Tc-nanocolloid in patients with other types of markers *in situ* or calcifications in the breast. These other non-radioactive markers do not affect the freehand SPECT technique.

The radiocolloid injection site for SLNB is still a matter of controversy [13,14,16,17]. The freehand SPECT method as described in this study is only of clinical relevance for tumour-related tracer administration. For injections in the vicinity of the tumour, this technique is sufficient. However, for injections in small lesions, this technique requires more precision. This could be acquired with an optically tracked needle integrated in the freehand SPECT system. There are prototypes of needles or catheters with optical tracking systems, enabling exact needle tip localisation and thereby possibly more accurate injections [26]. For the 36 freehand SPECT-guided injections, we used 15-, 25-, 35-mm needles, and the depth was determined on the basis of depth estimation.

### Optimisation of freehand SPECT

**Figure 5:**
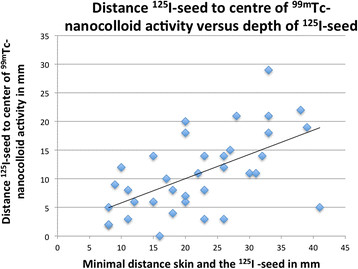
**Distance vs. depth.** Distance from ^125^I seed to the centre of the ^99m^Tc-nanocolloid activity versus the depth of ^125^I seed from the skin.

There are several possibilities to explain the observed distance deviation between the ^99m^Tc-nanocolloid injection depot and the ^125^I seed on SPECT/CT. First, the use of older (weaker in radioactivity) ^125^I seeds may give significantly less signal, which influences the image quality. Another explanation could be the fact that the freehand SPECT device indicates a depth and a direction, which are marked on the skin. The nuclear physician had to inject exactly similar to this direction, or else larger deviations in deeper injections would logically be the result. Further analysis of the relation of depth and inaccuracy hinted to a relation, where an increase in depth results in more inaccurate injections (correlation of 0.58) (Figure 5). When only the ^125^I seeds with a depth of <26 mm are taken into consideration (25/36 ^125^I seeds), the average distance between the ^99m^Tc-nanocolloid injection depot and the ^125^I seed is 8.2 mm (SD 5.1 mm, range 0 to 20 mm) This is less than the average distance measured on the SPECT/CT scans for all US-guided injections (group 2).

The retrospective evaluation of the accuracy in ten injections with higher number of measurements demonstrated a mean distance of 10.0 mm instead of 11.2 mm in the first 26 injections; this suggests a favourable relation to obtain more measurements. With these small numbers, this is not a significant conclusion. Nevertheless, we recommend using higher number of measurements, because more data for the calculations would logically result in more accurate reconstructions and could, for example, compensate the weak signal of older ^125^I seeds. An additional source of error in the evaluation might be the registration between CT and SPECT and the slice thickness of the CT images. These factors can have both positive and negative impacts on the evaluation but have to be considered when looking at the standard deviation of the results.

In the present study, freehand SPECT reconstruction was based on settings used for standard intraoperative procedures. In theory, it is possible to increase the number of iterations or reduce the voxel size. The standard number of iterations for reconstruction is 20; experimental settings where the number of iterations rises up to 100 iterations can result in more accurate localisations but may drastically increase the reconstruction time. The voxel size is also variable; this can be reduced from 5- to 2-mm voxels. Experimental setups will be required in the future to evaluate which are the optimal settings for specific applications. This study also demonstrates potential use of freehand SPECT for intraoperative ^125^I seed localisation since accurate navigation to the radioactive tumour marker is enabled. Furthermore, the margins of breast cancer specimens relative to the ^125^I seed could be determined *ex vivo* as predictor for surgical margins. A prospective study to investigate this assumption is currently in preparation.

## Conclusion

Peri-/intratumoural ^99m^Tc-nanocolloid injection for SLN mapping using freehand SPECT in patients with non-palpable breast tumours and implanted ^125^I seeds for tumour excision is feasible. This approach may become a reliable alternative for US-guided ^99m^Tc-nanocolloid injections, alleviating daily/clinical logistics in both nuclear medicine and radiology departments.

## Competing interests

This work was performed in the framework of the Eurostar's project ‘E!7103, real-time fhSPECT’. The authors declare that they have open access. This article is distributed under the terms of the Creative Commons Attribution Non-Commercial License which permits any non-commercial use, distribution, and reproduction in any medium, provided the original author(s) and source are credited.

## Authors' contributions

BP drafted the manuscript, substantially contributed to the conception and design of the study, and acquired, analysed and interpreted the data. LWV substantially contributed to the conception and design of the study and revised the manuscript critically for important intellectual content. DH and OB substantially contributed to the conception and design of the study and acquired clinical data. MVP drafted and revised the manuscript critically for important intellectual content. MS and RVO approved the final manuscript for submission and publication and revised the article critically for important intellectual content. All authors read and approved the final manuscript.
